# The skin-wrinkling test: principles and clinical applications

**DOI:** 10.1055/s-0046-1827046

**Published:** 2026-08-13

**Authors:** Otto Jesus Hernández Fustes, Cláudia Suemi Kamoi Kay, Paulo José Lorenzoni, Renata Dal-Prá Ducci, Paula Raquel do Vale Pascoal Rodrigues, Orlando Graziani Povoas Barsottini, Rosana Herminia Scola

**Affiliations:** 1 Universidade Federal do Paraná, Complexo do Hospital de Clínicas, Curitiba PR, Brazil.; 2 Universidade Federal do Paraná, Curitiba PR, Brazil.; 3 Universidade Federal de São Paulo, Departamento de Neurologia, São Paulo SP, Brazil.

**Keywords:** Autonomic Nervous System, Vasoconstriction, Small Fiber Neuropathy

## Abstract

Cutaneous wrinkling is a normal phenomenon that occurs on the palms in a reversible manner and results from vasoconstriction influenced by vasomotor function. It can be assessed through the skin-wrinkling test following water immersion, with the absence or reduction of wrinkling being associated with disorders affecting the dense network of sympathetic nerves in these regions. The literature suggests that this test may serve as a screening tool for small-fiber neuropathy, particularly in settings in which biopsy is not available. It is a rapid, inexpensive, and easily-accessible test. We herein review the principles of the skin-wrinkling test, its pathophysiology, and its clinical indications as a tool to evaluate the sympathetic component of the autonomic nervous system.

## INTRODUCTION

The skin-wrinkling test (SWT) is a simple yet physiologically-complex clinical method, non-invasive and widely used to assess postganglionic sympathetic autonomic function, with an emphasis on sudomotor innervation. By integrating aspects of autonomic neurophysiology, cutaneous microcirculation, and the biomechanical properties of the epidermis, the SWT holds particular relevance for the investigation of peripheral neuropathies, especially small-fiber neuropathy (SFN). 1 2

The SWT is based on the sympathetic vasomotor response of glabrous skin, particularly at the digital pulps. During immersion in warm water (40 °C), a reflex activation of the postganglionic cutaneous sympathetic nervous system occurs, leading to vasoconstriction of the digital arterioles through the release of noradrenaline and activation of Alpha-adrenergic receptors on vascular smooth muscle. This vasoconstriction reduces local blood volume and decreases hydrostatic pressure within the dermal capillaries, resulting in reduced tissue turgor. Consequently, the skin becomes less distended and forms superficial folds and wrinkles, especially in regions with a high density of sweat glands. 1 2

Thus, skin wrinkling does not represent mere water absorption by the epidermis, but rather an active neurovascular phenomenon that depends on the integrity of postganglionic sympathetic fibers responsible for vasoconstriction. This effect occurs only in glabrous skin, which lacks hair follicles and, in the palms and soles, contains the highest concentration of sweat glands in the body. 2

Several mechanisms have been proposed to explain skin wrinkling, including edema of the stratum corneum, which may be related to the pH difference between epidermal keratin and water. 2 3 4 In addition, an increase in water temperature itself leads to greater epidermal edema. 4 Other proposed mechanisms include increased turgor of deeper tissues and contraction of myoepithelial cells in the absence of sebaceous glands in glabrous skin. 2

Reduced or absent wrinkling after water immersion has been investigated across various clinical conditions, particularly in SFN, given that normal wrinkling depends on the functionality of intact peripheral nerves. 2 5 Accordingly, the SWT emerges as a useful tool for SFN screening, especially in settings in which skin biopsy is not available.

## METHODS

The current scoping review was conducted in accordance with the Preferred Reporting Items for Systematic Reviews and Meta-Analyses extension for Scoping Reviews (PRISMA-ScR). The methodological approach was chosen to comprehensively map the breadth of evidence regarding the physiological mechanisms, examination techniques, and clinical applications of the SWT. No review protocol was registered.

The primary objective of was to map and synthesize the existing literature on the SWT. The review was guided by the following research questions:

- What physiological mechanisms underlie water-induced skin wrinkling?
- How has the SWT been performed and graded across studies?
- In which clinical conditions has the SWT been investigated as a diagnostic or screening tool?
- What evidence supports its role as a marker of postganglionic sympathetic or small-fiber dysfunction?

Studies were eligible for inclusion if they met the following criteria: Human studies evaluating water-induced or pharmacologically induced skin wrinkling, experimental, physiological, observational, or clinical study designs, studies addressing autonomic function, sympathetic dysfunction, or peripheral and small-fiber neuropathies, original research articles, methodological studies, and relevant narrative or historical reviews, articles published in English, and no restriction on year of publication.

The following were excluded: animal-only studies, studies unrelated to autonomic or peripheral-nerve function, case reports without relevant physiological or methodological insights, and conference abstracts without the full text available.

A comprehensive literature search was conducted on the PubMed/MEDLINE database from inception through November 2025. The search strategy combined Medical Subject Headings (MeSH) and free-text terms, including: *skin wrinkling test* , *water-immersion wrinkling* , *small fiber neuropathy* , *autonomic neuropathy* and *sympathetic dysfunction* .

The titles and abstracts of all identified records were screened for relevance regarding the review objectives. Full-text articles were retrieved for records deemed potentially eligible. Final inclusion was determined after full-text assessment according to the predefined eligibility criteria.

## PHYSIOLOGY OF WRINKLING

Skin wrinkling after prolonged immersion in water is a normal phenomenon dependent on the sympathetic nervous system. It results from vasoconstriction, which is influenced by vasomotor function. 6 7

Cutaneous sympathetic innervation is provided by postganglionic sympathetic fibers (originating from T1–T3) that travel through peripheral nerves and reach the dermis and subcutaneous tissue. These fibers terminate in vasomotor plexuses that regulate arterioles and arteriovenous anastomoses. There are also fibers that innervate sweat glands and the arrector pili muscle, all under cholinergic or adrenergic control.

The primary neurotransmitter is noradrenaline, which binds to Alpha-1 receptors on vascular smooth muscle, producing vasoconstriction. This contraction reduces arteriolar caliber and blood flow; consequently, subcutaneous tissue turgor decreases, enabling the epidermis to fold—thus forming the characteristic wrinkles.

Heated water acts as a physical stimulus: it increases the diffusion and conductivity of the skin, facilitating reflex sympathetic activation. It stimulates sudomotor secretion, a functional marker of local autonomic integrity. In areas where sweat glands and sympathetic fibers are damaged, thermal stimulation does not elicit reflex vasoconstriction, and wrinkles do not form ( Table 1 ).

**Table 1:** Pathophysiological summary

Step	Physiological Event
**Stimulus**	Immersion in warm water → reflex sympathetic activation
**Neurotransmitter**	Release of noradrenaline (via postganglionic fibers)
**Vascular effect**	Vasoconstriction of dermal arterioles
**Hemodynamic result**	Reduction in capillary volume and subcutaneous turgor
**Cutaneous manifestation**	Formation of wrinkles due to epidermal collapse over retracted underlying tissue
**Dysfunction**	Sympathetic lesion → absence of vasoconstriction → skin remains smooth

Historically, it was believed that wrinkling resulted from osmotic absorption of water by the epidermis, causing irregular swelling. In healthy individuals, immersion of the forearms in warm water (43–45 °C) induces vasodilation in the lower extremities. 8

Classic studies 9 10 demonstrated that, in sympathetically-denervated areas (for example, following median or ulnar nerve injury), wrinkling does not occur even with the same water exposure. If the sympathetic nervous system is blocked (through local anesthesia or sympathectomy), wrinkling likewise disappears, thereby confirming that the mechanism is neurogenic rather than purely physical.

In their pioneering study, Lewis and Pickering 9 investigated the physiological mechanism responsible for fingertip wrinkling after water immersion, testing whether the phenomenon was caused by osmotic absorption or by neurovascular activity. Healthy volunteers underwent immersion of the fingers in warm (∼ 40 °C) and cold (∼ 20 °C) water. Observations were made regarding the timing and pattern of wrinkle formation, and in some cases local sympathetic blockade was induced via regional anesthesia, or participants with preexisting nerve injuries were examined. 9

They 9 observed an absence of wrinkling in anesthetized or denervated areas even after prolonged immersion. The phenomenon depended on the integrity of the sympathetic nervous system and its postganglionic fibers. Temperature influenced the rate of wrinkling: it occurred more rapidly in warm water, was absent in cold water, and resulted from vasoconstriction of the digital arterioles and the consequent collapse of the epidermis. Their findings demonstrated that digital wrinkling is an active sympathetic vasomotor response rather than osmotic water absorption by the epidermis. The study 9 established the physiological basis of the phenomenon and its relevance as an indicator of peripheral sympathetic integrity.

Lewis and Pickering 9 were the first to prove that digital wrinkling is an active sympathetic vasomotor response rather than the result of osmotic absorption. Their work established the concept that the wrinkling test can be used as a functional indicator of peripheral sympathetic integrity—an idea later revisited and expanded by Bull and Henry 10 and Wilder-Smith et al. 1 ( Table 2 ).

**Table 2:** Historical comparison

Aspect	Lewis and Pickering 9 (1936)	Bull and Henry 10 (1977)
Nature	Experimental; basic physiology	Clinically-applied
Objective	To understand the mechanism of wrinkling	To validate it as a functional test
Method	Experimental; nerve blockade and thermal variation	Observational; standardized immersion and visual grading scale
Conclusion	Active sympathetic vasoconstriction	Marker of postganglionic sympathetic function
Contribution	Establishment of the physiological basis	Clinical standardization of the test; development of a reproducible clinical protocol

In 1977, Bull and Henry 10 reevaluated the SWT as a clinical marker of postganglionic sympathetic function, establishing standardized parameters for its performance and interpretation. They studied healthy volunteers and patients with peripheral nerve lesions (to the median and ulnar nerves). The fingers were immersed in warm water (40–45 °C) for 30 minutes, and the degree of wrinkling was visually assessed using a scale from 0 to 4. In some cases, sympathetic blockade or local anesthesia was applied to observe the resulting effects.

They 10 observed:

- absence of wrinkling in denervated or anesthetized areas, confirming dependence on sympathetic innervation;
- preservation of wrinkling in regions with intact innervation;
- a correlation between the degree of wrinkling and the level of vasomotor function; and
- that the test was reproducible, simple, and low-cost, making it suitable for clinical use.

The authors 10 provided clinical validation of the SWT test as a functional assessment of postganglionic sympathetic integrity, defining standardized clinical parameters and reinforcing its diagnostic utility in neuropathies and dysautonomias. This study is considered the milestone that consolidated the test as a diagnostic tool for autonomic neuropathies and peripheral nerve injuries.

They 10 suggested that wrinkling depends on two sets of factors: epidermal and deep tissue.

- Epidermal factors: After immersion, the stratum corneum of the epidermis absorbs water and swells, the degree of swelling depending on several factors, including the facts that the greater the difference between the pH of the fluid and the pK of the epidermal keratin, the greater the swelling; the higher the water temperature, the greater the swelling; and the higher the concentration of sodium chloride in the environment, the greater the swelling.
- Deep tissue factors: Provided that the turgor of the deep tissues is sufficiently low, swelling of the epidermis will lead to wrinkling. Turgor is high when sympathetic tone is low and vessels are dilated. Thus, in sympathectomized hands, which are typically warm, dry, and swollen, the tissue turgor is too great to enable epidermal swelling to lead to wrinkling. Wrinkling will also not occur when there is sufficient edema.

The fact that wrinkling occurs only in glabrous skin suggests that anatomical differences in these regions play a role in sympathetic innervation. Since one of the functions of sympathetic innervation in the hand is the regulation of blood flow, changes in cutaneous circulation during immersion can be investigated. 3

In 2003, Wilder-Smith and Chow 3 proposed that immersion-induced skin wrinkling is directly related to vasoconstriction, such that the regions with the greatest reduction in blood flow—particularly the fingertips—exhibit the most pronounced wrinkling.

Moreover, the pores through which sweat is released are important for the occurrence of wrinkling, as glabrous skin lacking such pores—such as that of the penis and clitoris—does not wrinkle. Thus, the pore may serve as an entry point to initiate the process: because sweat ducts traverse the skin transversely to reach the sweat glands, they enable water to come into contact with the dense neuronal network responsible for vasomotor control. Water entering the ducts alters the electrolyte composition and leads to increased activation of nearby neurons. 3

Win et al. 11 prospectively studied 70 healthy individuals with no cardiovascular risk factors or neurological disease. All participants underwent heart rate variability and SWT studies, concluding that skin wrinkling is related to central autonomic function.

Kareklas et al. 12 investigated the adaptive function of water-induced finger wrinkling. The study tested the hypothesis that wrinkles improve the handling of wet objects by increasing friction and facilitating water drainage at the finger–object interface. Volunteers performed grasping and object-transfer tasks under three conditions: dry fingers, wet fingers without wrinkles, and wet fingers with wrinkles induced by immersion. The results showed that, when handling wet objects, the participants with wrinkled fingers were significantly faster than those with merely wet fingers, whereas no performance differences were observed when handling dry objects. The authors 12 concluded that finger wrinkling represents a functional adaptation that enhances grasping efficiency in wet environments, supporting the view that it is an active mechanism mediated by the autonomic nervous system rather than a passive consequence of water absorption by the skin.

## EXAMINATION TECHNIQUE

The SWT can be performed using water immersion or a eutectic mixture of local anesthetics (EMLA). 5 7 13

**Figure 1:**
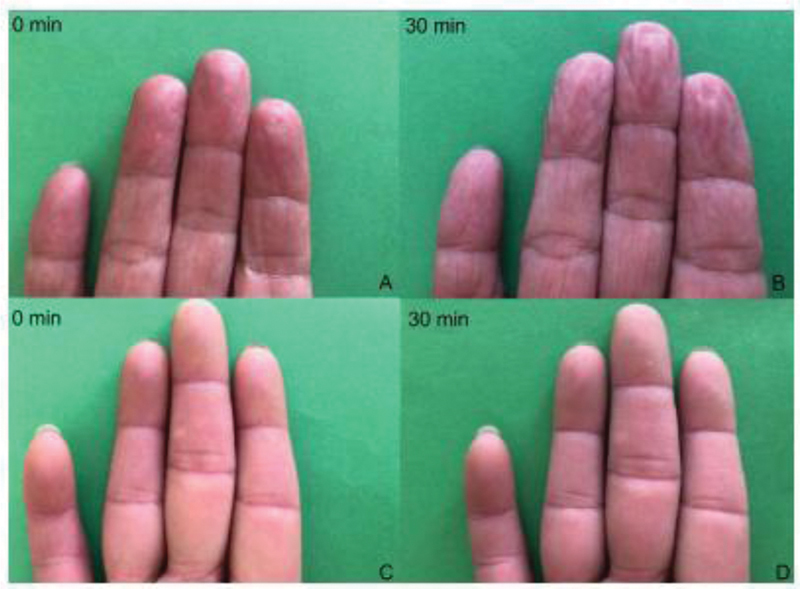
Normal skin-wrinkling test ( **A,B** ); abnormal skin-wrinkling test ( **C,D** ). 20

The SWT following immersion of the hand and forearm in water at 40 °C for 30 minutes ( Figure 1 ) was first described in 1973 by O'Riain, 14 who observed that denervated skin did not wrinkle. Water temperature control is maintained using a digital thermometer.

**Figure 2:**
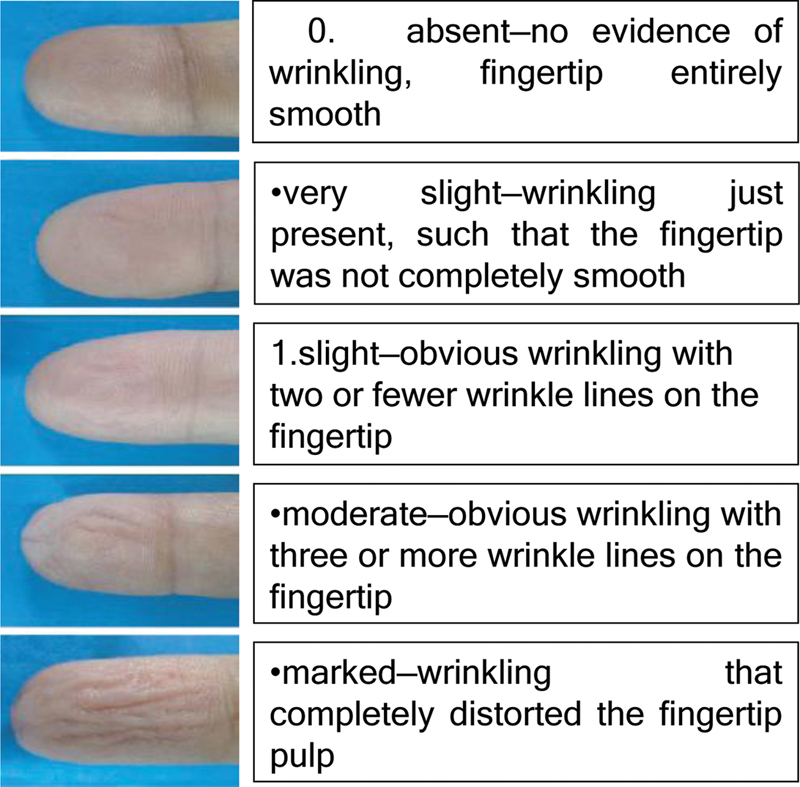
Stimulated wrinkling grading. 7

In 1984, Clark et al. 4 performed the SWT using water maintained at a constant temperature via a thermostat. Wrinkling was graded according to the following scale ( Figure 2 ):

0. absent—no evidence of wrinkling, fingertip entirely smooth;
1. very slight—wrinkling just present, such that the fingertip was not completely smooth;
2. slight—obvious wrinkling with two or fewer wrinkle lines on the fingertip;
3. moderate—obvious wrinkling with three or more wrinkle lines on the fingertip; and
4. marked—wrinkling that completely distorted the fingertip pulp.

Each finger could therefore be graded from 0 to 4, with a maximum score of 20 for each hand. The test was also performed on the feet; however, a wide variability in wrinkling patterns was observed, indicating a lack of standardization and thus rendering the examination unsuitable for the feet. 4

Teoh et al. 7 applied the wrinkling grading scale originally described by Clark et al. 4 The test was performed by immersing the patients' right hand in water at 40 °C containing 0.05 mmol/L of sodium chloride (NaCl). All digits except the thumb were graded. Normal wrinkling was defined as a score of 4 or higher.

Datema et al. 6 conducted the SWT using photographic documentation. Photographs of the patients' palms were taken before and after the test. Participants immersed their hands in water at 40 °C for 30 minutes. Wrinkling was graded as 0.0 for absent wrinkling, 0.5 for minimal wrinkling, and 1.0 for distinct wrinkling, based on a comparison with the pretest photographs. Minimal and distinct wrinkling were considered normal, whereas absent wrinkling was classified as abnormal.

## STUDIES INVOLVING THE SWT

Braham et al. 15 evaluated wrinkling in 12 patients before and after cervical sympathectomy for hyperhidrosis, in the absence of other neurological diseases. Before surgery, hands were immersed in water at 40 °C for 20 minutes, and wrinkling occurred in all cases. Two days after surgery, the skin showed no wrinkling in most patients, and only minimal wrinkling in a few cases.

Clark et al. 4 conducted a study including a control group of 100 participants (43 men and 57 women), aged 18 to 83 years, and a group of 42 diabetic patients (30 men and 12 women), aged 29 to 71 years. In the control group, there was no difference in wrinkling between the dominant and non-dominant hands, nor between men and women. Reduced wrinkling was observed among individuals engaged in manual labor. The mean wrinkling scores were: 18.6 ± 5.8 after 10 minutes; 25.0 ± 5.6 after 20 minutes; and 29.4 ± 6.3 after 30 minutes. The average number of wrinkled fingers in healthy participants was at least 9 out of 10, demonstrating that wrinkling occurs in most healthy individuals after 30 minutes of water immersion. 4

Among diabetic patients, wrinkling was significantly reduced compared with controls ( *p* < 0.001). 4 The degree of wrinkling did not correlate with disease duration or glycemic control. No difference was found between patients treated with insulin and those using oral hypoglycemic agents. Patients with symptoms of neuropathy had fewer wrinkled fingers (5.0 ± 3.5 versus 7.6 ± 2.7; *p* < 0.02). 4

Teoh et al. 7 assessed the effectiveness of the SWT as a diagnostic tool for SFN, comparing it with intraepidermal nerve fiber density (IENFD) and the sympathetic skin response (SSR). Patients with idiopathic SFN and healthy controls were studied. Wrinkling was evaluated after immersion in warm water (40 °C for 30 minutes) and after EMLA cream application, with results compared with IENFD and SSR. Abnormal wrinkling was found in 71% (water immersion) and 67% (EMLA) of the patients, correlating with reduced nerve fiber density. The SSR demonstrated lower sensitivity. The test proved simple, inexpensive, and comparable to skin biopsy for the diagnosis of small fiber neuropathy. 7

Wilder-Smith and Chow 3 evaluated 23 healthy individuals, measuring blood flow velocity in the ulnar artery and in the superficial digital vasculature of the fourth finger before and after immersion of the hands in a 0.5 mol/L NaCl solution at 40 °C for 30 minutes. Blood flow velocity significantly decreased after immersion across all tested vascular territories (12.8% in the ulnar artery, 23.2% in the digital arteries, and 7.4% at the skin surface of the fingertip pulp). 3

Van Barneveld et al. 16 confirmed the clinical feasibility of the test by studying healthy volunteers and patients with suspected dysautonomia. Wrinkling was visually assessed after finger immersion in water at 40 °C for 30 minutes. The study demonstrated good interobserver reproducibility, an influence of age and temperature, and highlighted the need for standardization of immersion time and grading scale. 16

Datema et al. 6 examined 35 patients with SFN, among whom 23 showed absent wrinkling. In contrast, among 61 healthy controls, 43 demonstrated some degree of wrinkling in both hands. The sensitivity of the SWT was of 66%, with an specificity of 70%.

Studies 6 13 have shown that the skin wrinkling index correlates with nerve fiber density in skin biopsies. The findings of Teoh et al. 7 suggest that the SWT0 should be used to screen for SFN, particularly in settings in which skin biopsy is not available. 7 The strongest evidence supporting the SWT as an indicator of sympathetic dysfunction in the limbs is its application in diabetic populations for the detection of diabetic neuropathy (sensitivity of 81.3% and specificity of 67%). 5

Ng et al. 17 evaluated patients with diabetes mellitus, with and without neuropathy, using the EMLA-induced SWT in comparison with nerve-conduction studies and vibration-threshold assessment. They concluded that the test has good sensitivity for distal diabetic neuropathy and performance comparable to that of standard diagnostic tests.

In a study of patients with dysautonomia and SFN using the digit-wrinkle scan to quantify digital wrinkling, Sopacua et al. 18 demonstrated that it is an objective and reproducible method to assess dermal sympathetic function.

## CLINICAL RELEVANCE AND APPLICATIONS

The SWT has gained increasing interest in the context of sSFN assessment, in which C and A-Delta fibers—which are responsible for thermal sensitivity, pain, and sudomotor function—are impaired. Because normal wrinkling depends on intact reflex mechanisms, the cutaneous response to immersion becomes a simple, low-cost functional marker. 2 3 19 20

Beyond SFN, the SWT has been investigated in a range of conditions, including diabetic autonomic neuropathy, dysautonomic syndromes, connective-tissue diseases, neurodegenerative disorders, and even in the monitoring of posttraumatic nerve recovery. 21 22 23 24 25

Its operational simplicity—requiring only a container with warm water and standardized clinical observation—makes the test particularly useful in resource-limited settings or as a complementary tool for initial screening.
